# Using Social Media to Promote Life Skills Among Adolescents: A Debate on Opportunities, Challenges, and Implications for Health and Education

**DOI:** 10.1007/s10935-025-00826-1

**Published:** 2025-01-28

**Authors:** Elizabeth Zimmermann, Samuel Tomczyk

**Affiliations:** 1 Department of Health and Prevention, Institute of Psychology, University of Greifswald, Robert-Blum-Str. 13, 17489 Greifswald, Germany; 2 German Center for Child and Adolescent Health (DZKJ), Partner Site Greifswald/Rostock, Greifswald, Germany

**Keywords:** Social media, Adolescents, Prevention and health promotion, Life skills, Media literacy

## Abstract

The digitalization of society increasingly blurs boundaries between analog and digital worlds, offering opportunities such as telemedicine and global connectivity through digital platforms. However, it also presents risks, including cyberbullying, addiction potential, harmful content, misinformation, and privacy concerns from data breaches and surveillance technologies. Social media, with its global reach, amplifies both opportunities for positive engagement and the responsibility to navigate largely unregulated content. Adolescents, due to their developing critical evaluation skills and high prevalence of mental health challenges, are particularly vulnerable in this space and must navigate the risks associated with social media while simultaneously leveraging it for essential developmental tasks, such as identity formation and building social connections. To support them in this challenge, we propose adapting the traditional life skills approach to address the challenges of social media use, empowering adolescents to navigate both digital and analog environments effectively. These life skills include critical thinking, communication, and managing emotions and stress – all of which are essential for navigating social media. Despite its complexities, social media offers a unique platform for health promotion and prevention interventions due to its wide accessibility and reach. It holds significant promise for adolescent health by providing information, motivation, and social support. However, evidence-based, youth-centered prevention strategies on these platforms are still in early development and require further research to ensure effectiveness. Prevention programs integrating proven behavior change techniques, aligning with the preferences and needs of adolescents, and teaching essential life skills can empower them to navigate digital and analog challenges effectively.

## Introduction

Digitalization has initiated a profound cultural shift, increasingly merging the digital and physical worlds, thereby creating new opportunities and risks. An important phenomenon in this context is the rise of social media, which is used by 5.17 billion people worldwide (Statista, 2024). Social media platforms allow users to create content and reach a wide audience (Korda & Itani, 2013). This rapid and boundless communication facilitates greater visibility of social, ethical, political, and environmental issues. While the unrestricted access to knowledge and the free dissemination of content offer many benefits, content is rarely censored or editorially reviewed, placing the responsibility for critical consumption and managing the associated impacts on the individual.

Adolescents, defined by the World Health Organization (WHO) as individuals aged 10 to 19 (WHO, 2001), are particularly in need of support in this regard. Despite being highly active in the digital realm, their digital, socio-emotional, and cognitive skills are often not yet fully developed to adequately navigate the challenges posed by social media use. These challenges include understanding complex digital mechanisms like algorithms, distinguishing between credible and non-credible sources, making moral decisions, and engaging beneficially in social interactions (Festl, 2021; Initiative D21, 2021). Additionally, adolescence is a period characterized by a high prevalence of mental health issues, with many psychiatric disorders first emerging during this stage of life (Bohl et al., 2022; Steffen et al., 2019).

In light of the widespread use of social media among adolescents and the associated opportunities and challenges, this article aims to explore ways to empower adolescents for healthy and beneficial social media use while strengthening their overall well-being, guided by the established concept of life skills.

## Opportunities of Social Media for Adolescents

Social media has become an integral part of adolescents’ daily lives and social environment. 95% of teens aged 13 to 17 use YouTube, making it the most widely used platform among this group. Other platforms such as TikTok (63%) and Instagram (59%) are also highly popular (Vogels et al., 2022). The amount of time spent on social media increases with age: 15- to 16-year-olds spend almost twice as much time on these platforms as 9- to 11-year-olds (Blanchard et al., 2023). In Germany, the average daily time spent on social media among adolescents in 2021 was 241 min, partly influenced by the COVID-19 pandemic (DAK, 2020). Social media provides adolescents with opportunities to interact with peers, explore their identities, practice self-presentation, and enhance autonomy, motivation, and decision-making skills (Moorhead et al., 2013; Nesi et al., 2018). Thus, they use social media for important developmental tasks. As a result, social media use can positively contribute to flourishing by enhancing relationships through social support and the development of social capital. It can also play a vital role in strengthening identity, particularly for minorities and individuals with disabilities, by providing spaces for representation and self-expression. Additionally, social media can foster a sense of mastery through opportunities for learning, building self-efficacy, and achieving personal accomplishments. Collectively, these experiences of connection, inspiration, and empowerment can promote positive well-being (Gudka et al., 2021).

## Risks of Social Media for Adolescents

While social media may promote social connection and provide a sense of belonging (Baumeister & Leary, 1995), they also present several risks: Meta-analyses show that the prevalence of social media addiction ranges from 5 to 31%, depending for example on the classification scheme used, cultural, geographical, and socioeconomical factors, or the design and features of the social media platform itself (Cheng et al., 2021; Ong & Lee, 2022).

Moreover, personal usage data is often sold to companies, and corresponding algorithms use this data to encourage consumption, which can undermine individual autonomy (Zuboff, 2019), with 72% of customer purchasing decisions being shaped by algorithms (Prabha et al., 2025). Furthermore, content is frequently shared without being regulated or censored according to youth protection standards, exposing users to harmful information and products (Arora et al., 2021; Dunlop et al., 2016). This can lead to the spread of misinformation, hate speech, and political polarization (Allcott et al., 2020). Almost 90% of young people between the ages of 18 to 34 have been exposed to harmful content at least once in their life (Enock et al., 2023). Additionally, users can become victims of cyberbullying or sexting (Kowalski et al., 2014; Rafla et al., 2014). According to a recent study, 55% of students in the United States aged 13 to 17 have experienced cyberbullying at some point in their lives (Cyberbullying Research Center, 2024).

Adolescents particularly need to be protected from the risks of social media use because their neural maturation processes are incomplete during the teenage years, and their knowledge of social media functions and legal regulations is often limited (Crone & Konijn, 2018; Initiative D21, 2021). However, not all adolescents are at the same risk regarding social media use and its consequences. In fact, social media use is influenced by various factors, such as emotion regulation, personality traits, and specific social needs, such as the need for belonging or the fear of missing out (Beyens et al., 2016; Ostendorf et al., 2020; Wartberg et al., 2021). When social media use exhibits addiction-like behaviors, such as preoccupation, compulsion, and excessive engagement, it is referred to as *problematic social media use* (Shannon et al., 2022). Recent meta-analyses and systematic reviews show that this dysfunctional way of using social media is associated with mental health problems like depression, anxiety, and loneliness, increased stress, and risky (sexual) behavior in adolescents with small to medium effect sizes (Huang, 2022; Leo et al., 2023; Shannon et al., 2022; Vannucci et al., 2020). In view of the many challenges, risks, and potentially negative symptoms associated with social media use, developing skills for a balanced social media use that considers risks and benefits can therefore be seen as both a central age-appropriate challenge and a key resource in the lives of adolescents (Festl, 2021).

## Life Skills in the Digital Era or Digital Life Skills

To this end, we propose integrating the established concept of life skills, essential for healthy development (WHO, 1999), with digital skills (Aufenanger, 2002; Brandhofer & Wiesner, 2018; Festl, 2021; Initiative D21, 2021), which are crucial for the autonomous use of digital tools like social media, into a unified framework of digital life skills.

Originally, life skills were defined as the abilities that enable individuals to effectively manage the challenges of everyday life. In 1999, the World Health Organization (WHO) identified five core areas of life skills: (a) decision making and problem solving, (b) creative and critical thinking, (c) communication and interpersonal skills, (d) self-awareness and empathy, and (e) coping with emotions and stress (WHO, 1999). Life skills promote academic success, health, and well-being (Hage et al., 2007; WHO, 1994, 1999) and are associated with the reduction of risky behaviors such as alcohol and drug abuse (Botvin, 1983) and bullying (Gaffney et al., 2019). They also contribute to the prevention and management of chronic diseases (Norris et al., 2001), positively affect the current and future mental health of children and adolescents, and serve as a key protective factor against mental disorders or health issues (Ciarrochi et al., 2003; Jones et al., 2015).

## Fostering Digital Competence and Life Skills

Life skills are also embedded in concepts such as (social) media literacy (Festl, 2021) and media competence (Aufenanger, 2002; Brandhofer & Wiesner, 2018), which have emerged with the rise of digitalization. These concepts encompass self- and social competence, critical thinking, and communication skills related to media, as well as the development of a media identity and corresponding self-awareness (Aufenanger, 2002; Brandhofer & Wiesner, 2018; Festl, 2021). Additionally, the Digital Skills Gap Index (Initiative D21, 2021) – a global ranking that assesses how well economies are prepared to meet existing and emerging digital challenges – emphasizes that people especially need problem-solving and reflection skills to understand and engage with digital mechanisms.

The strong overlap between life skills and digital skills highlights that, in today's digital world, it is necessary to master both. Young people face a multitude of challenges that manifest in both digital and analog forms (e.g., peer pressure, self-expression). Therefore, fostering life skills follows an integrative logic by focusing on the commonalities of these challenges in a digitally shaped world and how they influence human experiences and behavior. An overview of the necessity of life skills in the context of social media is provided in Table 1.

**Table 1:** Life skills (according to WHO, 1999) in the context of social media

Life skills	Examples for the necessity in the social media context
(a) Decision-making and Problem Solving	Decision-making is required in the process of the presentation of sensitive content as well as privacy settings. What content can securely be posted? What settings help protect privacy? Problem-solving skills are required when it comes to dealing with online problems such as cyber bullying, sexting, or other interpersonal conflicts. Once a dysfunctional way of using social media is established, people need problem-solving skills in order to counter their problematic use of social media.
(b) Creative Thinking and Critical Thinking	Creative and critical thinking is crucial when it comes to finding solutions for (interpersonal) problems that arise online, as well as handling with (health-related) information. People need to critically reflect on the relevance and truthfulness of content on social media. Creative thinking is further required to design a unique self-representation and identity online.
(c) Communication and Interpersonal Skills	Appreciative communication in the context of social media, such as recognizing, valuing, and positively reinforcing others' contributions, is crucial in fostering connection and mutual respect in digital interactions. Interpersonal skills are further required to deal with comments, opinions, peer pressure, trends etc. on social media. Maintaining social relationships online has further become a relevant skill in a more digitalized world.
(d) Self-awareness and Empathy	Building a feeling of self-worth and a strong identity online is a relevant step in establishing healthy self-awareness, as well as the functional reception of self-esteem-related content (body schema, idealized content). Empathy is required when interacting with social contacts online.
(e) Dealing with emotions and stress	Social media can be used in a dysfunctional way as an escape from reality. Healthy emotion and stress regulation is therefore crucial in order to be able to use the benefits of social media and protect oneself from the associated risks. The emotions that arise through use must also be functionally regulated.

## Opportunities of Social Media for Prevention and Health Promotion

Digital life skills offer a way to empower children and adolescents for competent use of social media. At the same time, these skills are relevant for addressing traditional lifestyle risks and developmental tasks. The fact that social media is a part of young people’s daily lives can even be advantageous for prevention and health-related purposes: Social media can serve as an effective medium for behavior change interventions by leveraging its interactive features, such as content sharing, quizzes, and social support, to implement behavior change techniques in engaging and accessible ways (Moorhead et al., 2013; Simeon et al., 2020). Platforms like Instagram and YouTube enable the dissemination of information, self-monitoring, and social processes that promote behavior change while catering to specific target group preferences (Vogels et al., 2022; Yardley et al., 2016). The selection of suitable applications and features must be guided by evidence-based strategies, target audience habits, and current platform functionalities to optimize engagement and impact (Arigo et al., 2018; Elaheebocus et al., 2018). In doing so, social media interventions can provide an effective alternative to conventional approaches by overcoming common barriers such as lack of health insurance and long wait times (Plaisime et al., 2020; Robards et al., 2019). Additionally, social media is very cost-effective and widely accessible, making it usable regardless of social or demographic factors (Moorhead et al., 2013). Digital platforms also offer flexible and continuous support, and they can be integrated into daily routines, increasing participant engagement and reducing dropout rates (Welch et al., 2016). In this way, interventions delivered through social media can promote physical, sexual, and mental health, encourage responsible use of alcohol, drugs, and the internet, and support preventive measures (Guse et al., 2012; Kruzan et al., 2022; Laranjo et al., 2014; Yonker et al., 2015).

When it comes to adolescents, research suggests that social media has the potential to profoundly reach young people and positively influence their health behaviors by providing information, motivation, and social support, while addressing them in their daily routines (Dudley et al., 2018; Gudka et al., 2021). However, findings on using social media for health and skill building among adolescents are very limited and inconsistent. Many studies focus on the digital footprints generated by social media use, which can provide valuable insights into people's mental states (Sultan et al., 2023). Some approaches successfully leverage apps to develop life skills and promote health (Gabrielli et al., 2020; Lampert, 2018; Paz Castro et al., 2022); however, the integration of life skills with social media platforms remains largely unexplored. In contrast, current discussions around the use of social media are often dominated by practical and ethical challenges, while insufficient attention is given to establishing clear theoretical and empirical links between social media and proven behavior change techniques (Martin et al., 2020; Plaisime et al., 2020). The challenge, therefore, lies in developing evidence-based preventive measures that respond to young people’s preferences while meeting scientific standards.

## Promoting Equality Through the Use of Social Media

Social media also presents both a challenge and an opportunity in terms of equality: While the internet makes information easily accessible, social inequality now results from the level of competence with which the internet in general and social media in particular are consumed and received (so-called *digital divide*) (Bittlingmayer et al., 2020; Initiative D21, 2021). Children from socially disadvantaged backgrounds are more likely to use social media both intensively and maladaptively, which increases their risk for psychosocial problems (Chung & Lee, 2019; DAK, 2020). Furthermore, socioeconomic inequality exacerbates health problems among many adolescents, as they often face more health issues while having less access to healthcare services (Quon & McGrath, 2014; Reiss et al., 2019). Moreover, in traditional life skills interventions, parental education levels and the social status of learners significantly influence the success of such interventions (Bittlingmayer & Hurrelmann, 2006). Given the high popularity and intensity of social media use among socially disadvantaged youth, and the corresponding high need for competencies for functional use – along with the low barriers to access – social media-mediated interventions could help improve equality of opportunity. Reaching these at-risk young people is particularly important and can be achieved through targeted advertisements on social media platforms or by collaborating with relevant schools and educational programs.

## Conclusion

In conclusion, social media, as an integral part of daily life, especially for children and adolescents, presents both challenges and opportunities for prevention and health promotion. Young people are exposed to numerous risks on social media, but they also spend considerable time there and can benefit from its potential. Therefore, social media offers a promising approach to reach adolescents with prevention and health promotion measures, providing versatile, low-threshold, and cost-effective interventions. In this context, digital life skills represent a concept that focuses on the commonalities between analog and digital challenges in the human experience. It also takes into account the developmentally sensitive phases of target groups and the need for age-appropriate strategies to achieve skill building. To foster the development of functional social media use, the risks associated with social media should be integrated into the development of interventions and considered in their implementation.

While the potential of using social media for health promotion is promising, this approach is not intended to replace existing methods. Instead, it can be seen as a complement in the field of prevention. Particularly in low-threshold, everyday interventions, social media offers possibilities that go beyond traditional methods, which can significantly enhance universal prevention strategies. However, this theoretical position requires empirical validation and refinement. Research on social media-mediated prevention – particularly among adolescents – is scarce and therefore urgently needed. Interventions should be designed with the target group in mind, leveraging insights into existing platforms and features to align with their preferences and address their specific needs. Innovative approaches are also needed to motivate young people to participate in these interventions.
